# The Effects of Oxygen Functional Groups on Graphene Oxide on the Efficient Adsorption of Radioactive Iodine

**DOI:** 10.3390/ma13245770

**Published:** 2020-12-17

**Authors:** Qian Zhang, Yangyang Gao, Zhanglian Xu, Sheng Wang, Hisayoshi Kobayashi, Jie Wang

**Affiliations:** 1 Shaanxi Key Laboratory of Advanced Nuclear Energy and Technology, and Shaanxi Engineering Research Center of Advanced Nuclear Energy, School of Nuclear Science and Technology, Xi’an Jiaotong University, Xi’an 710049, China; imzhangqian@stu.xjtu.edu.cn (Q.Z.); gyy2019@stu.xjtu.edu.cn (Y.G.); 2 Department of Chemistry and Materials Technology, Kyoto Institute of Technology, Matsugasaju, Sakyo-ku, Kyoto 606-8585, Japan; hisabbit@yahoo.co.jp

**Keywords:** iodine adsorption, DFT method, oxygen-containing functional group, graphene oxide

## Abstract

Oxygen-containing functional groups tend to induce a strong interaction between solid adsorbents and iodine molecules, yet have not been systematically investigated. Herein, on the basis of a series of nitric acid-treated graphene oxide (GO) with different contents of oxygen functional groups for iodine adsorption, it was found that the iodine uptake capacity is proportionate to the oxygen content and the diversities of oxygen-containing groups. The density functional theory (DFT) calculation results also suggest that oxygen-containing groups result in strong interactions between iodine molecules and the adsorbents through a covalent bond-forming process, among which -OH groups possess a higher adsorption energy averagely. Such theoretical and experimental work deepens our understanding of the effects of oxygen functional groups on iodine adsorption and provides novel ideas for future design and synthesis of high-performance solid adsorbents for radioactive iodine.

## 1. Introduction

Nuclear power has made great contributions to the society due to the high-power density and low greenhouse-gas emission [1,2]. However, the nuclear industry is always accompanied by the production of nuclear waste (e.g., spent nuclear fuel) or hazardous environmental release [3,4]. Several radioactive gases (^129^I, ^131^I, ^85^Kr, and ^127^Xe) are formed during the fission process [5]. Among them, ^129^I and ^131^I (half-lives being ~1.57 × 10^7^ years and 8.02 days, respectively) will be involved in the metabolic process of human and eventually cause thyroid injury [6,7]. Therefore, these two iodine radioisotopes must be captured efficiently and stored securely. Up to date, loads of adsorbents including silica [8], chalcogen-based aerogels [9], activated carbon [10], zeolites [11,12] and porous frameworks [13,14,15,16] have been studied during the last few decades.

In order to improve the surface chemistry of solid adsorbents for higher iodine uptake, heteroatom doping (e.g., nitrogen doping) has drawn great attention since their adsorption capacity can be effectively strengthened through efficient surface modification [17,18,19,20]. Recently, Kangxin Xiao et al. has successfully synthesized N-doped porous carbons from orange peel waste, the iodine adsorption capacity of which was up to 2252 mg·g^−1^ [10]. Meanwhile, we noticed that oxygen doping might have similar effects as well. For example, Deng et al. studied the surface features and adsorption behaviors of adsorbents from sludge to methylene blue and iodine, and the X-ray photoelectron spectroscopy (XPS) suggested that the functional groups with high contents of oxygen on the surface of adsorbent served as active sites for the adsorption process [21]. S. S. Zyryanov et al. attributed the most intense line at 529.6 eV in the XPS spectra to a chemical bond between oxygen and iodine during the investigation of a stainless-steel surface irradiated with protons in an iodine medium. They believe that one of the forms of iodine is also due to the O-I bond [22]. During the systematic investigation of the adsorption characteristics of radioiodine molecules on three kinds of low-index surfaces of Cu_2_O, the calculation was carried out using first principles density functional with periodic slab models. For the Cu_2_O (100) surface, it is unusually found that I_2_ molecule moves to the nearby O_s_ position (denoted as O_s1_) after being optimized in the case of Cu_s_ site, indicating that the adsorption of I_2_ molecule by surface oxygen atom is prior to that of copper ones [23]. Debasis Banerjee et al. reported two microporous metal organic frameworks for I_2_ adsorption. Both the single-crystal X-ray diffraction and Raman spectroscopy reveal distinct sorption sites of molecular I_2_ to the phenyl- and phenol-based linkers stabilized by the I···π and I···O interactions, which favors the selective uptake of iodine over water vapor [24].

With the characteristics of easy preparation, good chemical and thermal stabilities, porous carbon possessing large surface area and high porosity has drawn a great deal of attention as an ideal adsorbent for iodine capture. Moreover, carbon materials have shown the possibility of overcoming the weak interaction with the iodine gases through surface functionalization. Thus, a series of oxygen-rich microporous activated carbons were synthesized in our previous work [25]. A positive correlation was found between the iodine uptake value and the oxygen content in the iodine adsorption experiments. The sample with the highest oxygen content displayed an iodine capture capacity up to 6.44 g·g^−1^, preliminarily suggesting the effects of oxygen. However, apart from oxygen-containing functional groups (-OH, -COC, C=O and -COOH), there are several influencing factors including the specific surface areas (SSAs), pore size and pore volume, related to the iodine adsorption performance of porous carbon materials. More complicatedly, the textural properties such as SSA and pore volume vary according to the different oxygen content on the carbon materials [25,26]. Variability in both oxygen content and pore size is not ideal for studies aimed at determining the effect of the former. As a result, experiments and calculations in this field have not been done or reported before. Therefore, in this study we developed a series of nitric acid-treated samples based on graphene oxide, which were used to investigate the impact of oxygen content since the previously mentioned factors have negligible effects on them. To further reveal the interactions between oxygen-containing functional groups and iodine molecules, density functional theory (DFT) calculations were also performed, which prove the experimental results theoretically. Both the experiment and calculation results offer a new insight for the future design of solid adsorbents for iodine adsorption.

## 2. Materials and Methods

### 2.1. Material Synthesis

Graphene oxide (GO) was prepared by chemical exfoliation of graphite according to an improved Hummers method, which was then dispersed by sonochemical irradiation. Then, 1 g of 2000 mesh natural graphite, 34 mL of H_2_SO_4_ and 0.74 g NaNO_3_ were mixed together, which was then stirred constantly in an ice bath. Next, 5 g KMnO_4_ was added gradually while stirring. The temperature of the mixture was kept below 20 °C throughout the whole process by cooling. Subsequently, the mixture was stirred at 35 °C for 3 h and diluted with deionized water (250 mL). The color of the mixture changed into bright yellow after 4 mL of 30% H_2_O_2_ was added. The mixture was then ultrasonically treated for 1 h. The possible residual metal ions were removed by filtering and washing with 1 L of 1:10 HCl aqueous solution, which was then removed by 1 L of deionized water. The GO suspension was freeze-dried before being used.

The frozen GO (400 mg) were dissolved in 40 mL of nitric acid (HNO_3_ 68%), and a homogeneous mixture was obtained after 30 min sonication. After stirring for 1 h intensely, the mixture was transferred into a 50 mL Teflon-lined autoclave, sealed and heated at 100 °C, 110 °C and 120 °C for 6 h and the samples were labeled as GO-100, GO-110 and GO-120, respectively. The suspend solution was cooled to room temperature naturally. The final product was collected by centrifuging the mixture, and then dried in vacuum for further use after being washed with deionized water for multiple times.

### 2.2. Characterization

A CHN elemental analysis was performed using a vario EL cube elemental analyzer (Elementar, Langenselbold, Germany). The surface structure of the sample and energy dispersive spectroscopy (EDS) was obtained by a GeminiSEM 500 instrument (Carl Zeiss, Oberkochen, Germany). Transmission electron microscopy (TEM) was performed on a JEOL JEM-F200 (HR) instrument (JEOL LTD, Tokyo, Japan). The chemical state of the samples was analyzed by X-ray photoelectron spectroscopy using an AXIS ULtrabld instrument (Kratos, Manchester, UK). High-resolution spectra were charge corrected to the C 1s peak at 284.6 eV.

### 2.3. Iodine Adsorption Measurements

Approximately 20 mg of GO/GO-100/GO-110/GO-120 and excess amounts of iodine crystals were placed in two 5 mL beakers, respectively. The beakers were then transferred into a sealed glass vessel. The adsorption experiment was carried out at 75 °C under normal pressure. The adsorption amount was monitored by recording the sample mass as a function of time. The uptake capacity Q_t_ was calculated using Equation (1), in which m_0_ is the initial mass of GO/GO-100/GO-110/GO120, and m_t_ is the mass after t minutes of adsorption.

Qt = (m_t_ − m_0_)/m_0_ (1)

### 2.4. Calculation Method

DFT calculations were carried out using Gaussian09 program (Gaussian, Wallingford, CT, USA). The B3LYP functional is one of the most popular and accurate functionals. The 6-311G (d, p) basis sets were used for H, C and O atoms. For I atoms, the LANL2DZ, i.e., Los Alamos effective core potential together with double valence set (D95V) was employed.

## 3. Results and Discussion

### 3.1. The Morphology of GO after HNO_3_ Treatment

**Figure 1:**
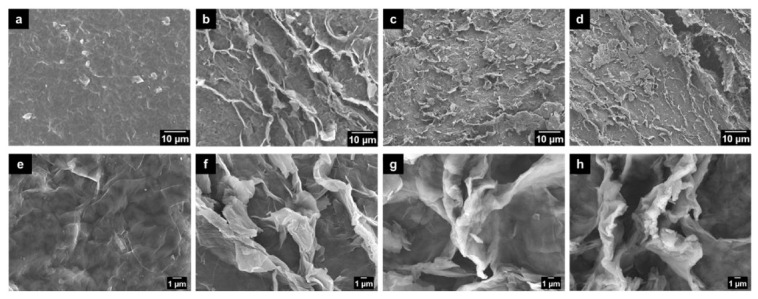
SEM images of (**a**,**e**) graphene oxide (GO), (**b**,**f**) GO-100, (**c**,**g**) GO-110 and (**d**,**h**) GO-120 with different magnification.

**Figure 2:**
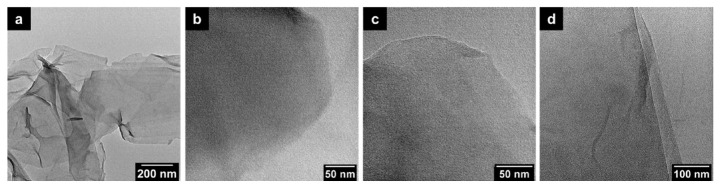
TEM images of (**a**) GO, (**b**) GO-100, (**c**) GO-110 and (**d**) GO-120.

The morphology of the samples was first studied via SEM. As demonstrated in Figure 1a,d, the surface of the original graphene oxide is relatively smooth. After HNO_3_ treatment by a simple hydrothermal reaction, the surfaces of all the samples obtained at different temperatures (100 °C, 110 °C and 120 °C) become rough (Figure 1b–d,f–h). A large number of wrinkles and cracks are distributed unevenly on the observed surfaces, which may be due to the strong oxidation effect of HNO_3_. With the temperature further increasing to higher levels, the cracks and wrinkles on the surface (e.g., GO-120) became more evident. In addition, transmission electron microscopy (TEM images in Figure 2b–d) reveals that the HNO_3_-treated graphene oxide remains the same as untreated graphene oxide nanosheets (Figure 2a) in the microscopic morphology despite the change in macroscopic morphology after HNO_3_ treatment.

### 3.2. Oxygen Functional Groups

**Figure 3:**
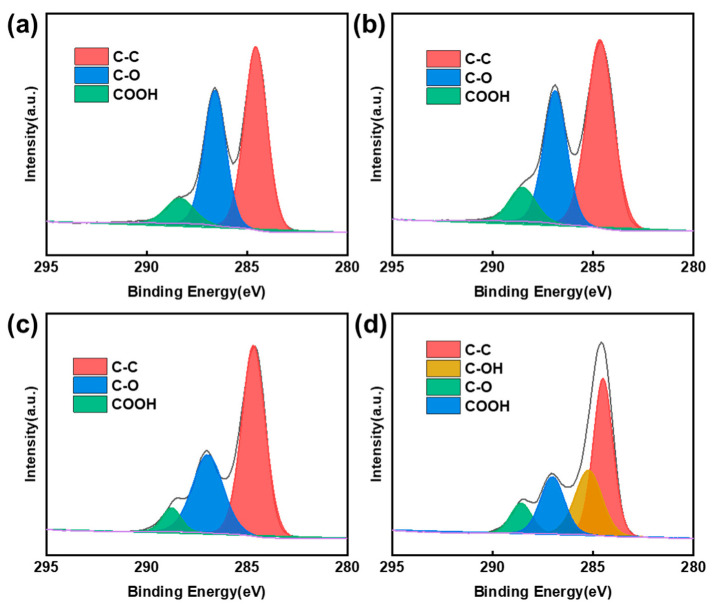
XPS C 1s analysis of (**a**) GO, (**b**) GO-100, (**c**) GO-110 and (**d**) GO-120.

**Figure 4:**
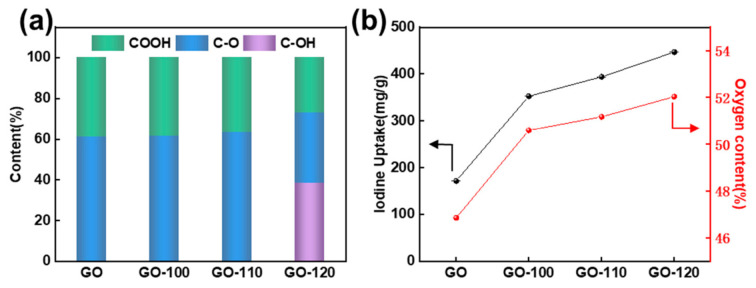
(**a**) contents of different oxygen-containing groups and (**b**) iodine uptake performance of GO, GO-100, GO-110 and GO-120.

The elemental compositions of the samples were further measured by CHN analysis. Table 1 shows the elemental content of all the samples. According to the results, the oxygen content of GO was the lowest (48.86 wt.%) and showed a slight upward trend after HNO_3_ treatment. More specifically, the oxygen content increased from 50.60 wt.% to 52.04 wt.% as the temperature increased from 100 °C to 120 °C. Similarly, the atomic ratio of C/O decreased from 1.51 to 1.23, indicating the existence of rich oxygen content in the HNO_3_-treated GOs. To further verify the intrinsic properties of the oxygen, the X-ray photoelectron spectroscopy was performed. The spectra were charge corrected (C 1s at 284.6 eV) and peak fitting of the high-resolution XPS spectra was also carried out, making it possible to estimate the bonding state of carbon and oxygen. The C 1s peaks of GO (Figure 3a), GO-100 (Figure 3b) and GO-110 (Figure 3c) can be assigned to three peaks being C-C (284.6 eV), C-O (286.6 eV) and COOH (289.0 eV), respectively [27,28,29]. For the sample GO-120 (Figure 3d), in addition to the three peaks mentioned above, one more peak appears at the binding energy of 285.2 eV, which can be attributed to C-OH groups [30,31,32]. Figure 4a shows the contents of different oxygen-containing surface functional groups on GO, GO-100, GO-110 and GO-120 calculated by fitting the C 1s peaks in Figure 3. There are two types of functional groups on the surface of GO/GO-100/GO-110, COOH and C-O. After HNO_3_ treatment, the content of C-O group shows a slight increase. When the temperature of hydrothermal reaction further reaches 120 °C, both the COOH and C-O group content decreased moderately, while the C-OH group accounts for approximately 40%.

### 3.3. The Iodine Adsorption Performance

**Figure 5:**
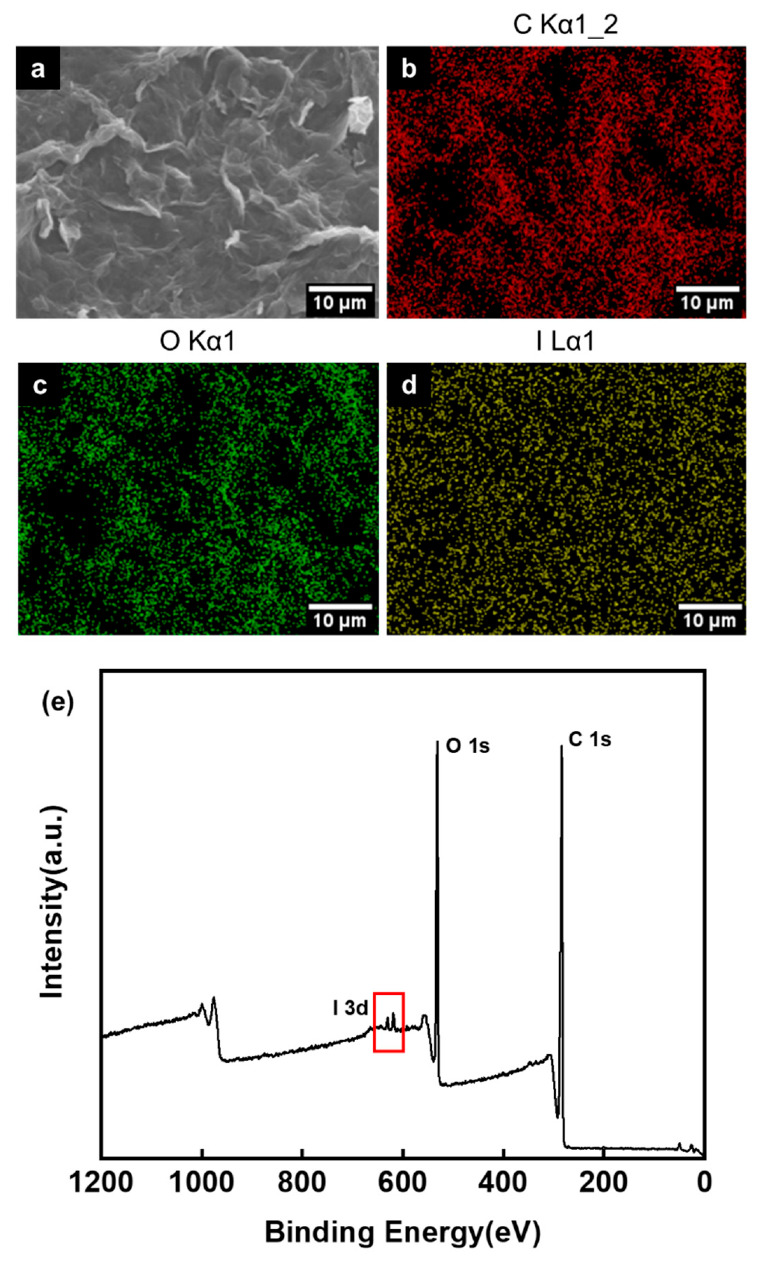
(**a**) SEM image, (**b**–**d**) elemental mapping images and (**e**) XPS wide scan spectrum of GO-120 after iodine adsorption.

Considering the high oxygen content and the diversity of surface functional groups, the obtained sample was used as a solid adsorbent for iodine vapor capture. The adsorption measurements were performed in a sealed vessel at 75 °C under atmospheric pressure [27,33]. The adsorption amount was monitored by recording the sample mass as a function of time. Figure 4b shows the iodine adsorption performance of the four samples. Although the saturated iodine adsorption capacity of the HNO_3_-treated GOs was not as high as that of the oxygen-rich activated carbon with ultra-high surface area used as adsorbent in our previous work (6.44 g·g^−1^) [25], the relationship between the capture value and the oxygen content is evident in Figure 4b. The iodine capture capacity increased from 0.17 g·g^−1^ to 0.44 g·g^−1^ proportionally with increasing oxygen content (Table 1). The GO-120 shows the highest saturation value. Figure 5 shows the EDS and XPS results of GO-120 after adsorption of iodine. The elemental mapping displayed in Figure 5c,d proves its oxygen-rich nature and confirms the successful adsorption of iodine on it. Figure 5e shows the wide scanning XPS spectrum of GO-120 after iodine adsorption. It can be seen that in addition to the two peaks corresponding to C 1s and O 1s, I 3d peaks are also present in the spectrum, again illustrating the same point. 

### 3.4. DFT Calculation

Based on the above experimental results, the positive influence of high oxygen content deriving from the abundant oxygen-containing surface functional groups for the iodine adsorption performance can be confirmed. To further deeply reveal the effects of different oxygen functional groups, the interactions between the carboxyl group (-COOH), hydroxyl group (-OH), epoxy group (-COC) and carbonyl group (-C=O) on the carbon surface and iodine molecules were studied via density functional theory calculations. In the calculations, two models (the perfect model and the defected model) were performed. Firstly, perfect graphene surfaces were considered. All the oxygen-containing functional groups were placed on the edged and central position over the graphene surface (Figure S1, Supplementary Materials). On the perfect graphene surface, -COOH group and -C=O were only placed on edged position (perfect-edged-COOH and perfect-edged-C=O) and -COC group was only placed on central position (perfect-central-COC) due to the instability issue. Due to the impossibility of centrally located structure of COOH, the addition of -COOH group around perfect-central-OH group and around perfect-central-COC was not considered [34].

**Figure 6:**
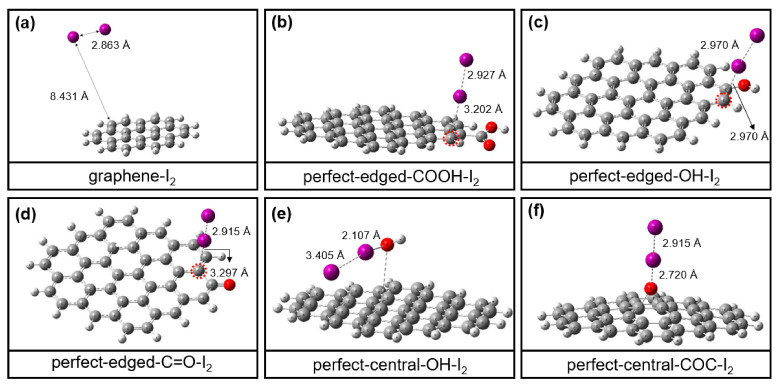
B3LYP functional-optimized structures for (**a**) graphene-I_2_ (C_24_H_12_-I_2_), (**b**) perfect-edged-COOH-I_2_, (**c**) perfect-edged-OH-I_2_, (**d**) perfect-edged-C=O-I_2_, (**e**) perfect-central-OH-I_2_ and (**f**) perfect-central-COC-I_2_ systems.

**Figure 7:**
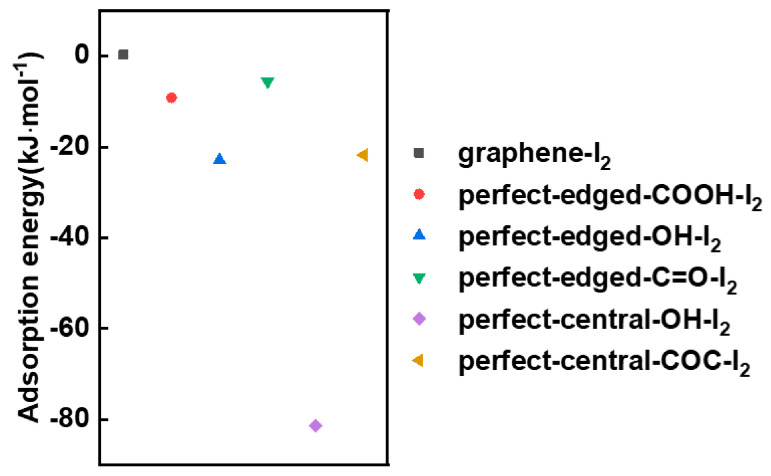
Adsorption energy of graphene-I_2_ (C_24_H_12_-I_2_), perfect-edged-COOH-I_2_, perfect-edged-OH-I_2_, perfect-edged-C=O-I_2_, perfect-central-OH-I_2_ and perfect-central-COC-I_2_ systems.

Figure 6 shows the B3LYP functional-optimized structures for graphene-I_2_ (C_24_H_12_-I_2_), perfect-edged-COOH-I_2_, perfect-edged-OH-I_2_, perfect-edged-C=O-I_2_, perfect-central-OH-I_2_ and perfect-central-COC-I_2_ systems. The corresponding adsorption energies are summarized in Table S1 and plotted in Figure 7. Figure 6a shows the interaction between I_2_ and pure graphene (the C_24_H_12_ model) without any oxygen functional groups. As a result, the I-C distance is very long (8.431 Å), and the I-I bond distance is unchanged compared with the I-I distance in an isolated I_2_ molecule (2.863 Å). Accordingly, the adsorption energy is estimated to be +0.3 kJ·mol^−1^ (unstable), suggesting a weak interaction between iodine molecule and graphene. In comparison, when iodine molecules are exposed to the oxygen-containing graphene surfaces, the I-I bond distances become longer in all cases (Figure 6b–f and Table S2), indicating the iodine molecules were attracted to the oxygen-containing groups. Interestingly, it’s found that iodine molecules would bind not only with oxygen atoms but also with carbon atoms activated by nearby oxygen functional groups (within red circles). For example, three carbons atoms were calculated to coordinate with the iodine molecule with the bond distances of 3.202 Å in perfect-edged-COOH-I_2_ system, 2.970 Å in perfect-edged-OH-I_2_ system and 3.297 Å in perfect-edged-C=O-I_2_ system, which are much shorter than that of I-C distance between I_2_ and pure graphene. It means that the introduction of the oxygen functional groups on the graphene surface would activate the nearby carbon atoms, enhancing the interaction between I_2_ and graphene. Meanwhile, the bond distances between iodine molecules and two oxygen atoms in perfect-central-OH-I_2_ system and perfect-central-COC-I_2_ system were calculated to be 2.107 Å and 2.720 Å, respectively. Accordingly, the binding energy of I_2_ on surface oxygen functionalized graphene became negative approximately from −9.2 to −81.4 kJ·mol^−1^ (Figure 7), indicating the enhanced adsorption of I_2_ on the oxygen functionalized graphene compared to that on the graphene (C_24_H_12_ model). Notably, the binding energy of I_2_ on perfect-central-OH reached a high of −81.4 kJ·mol^−1^, which is much larger than other oxygen functional groups, showing the strongest interaction between I_2_ and OH-terminated graphene. This calculation result is well consistent with the experimental results of the highest iodine capture for GO-120, where GO-120 also contains more -OH than the other samples.

**Figure 8:**
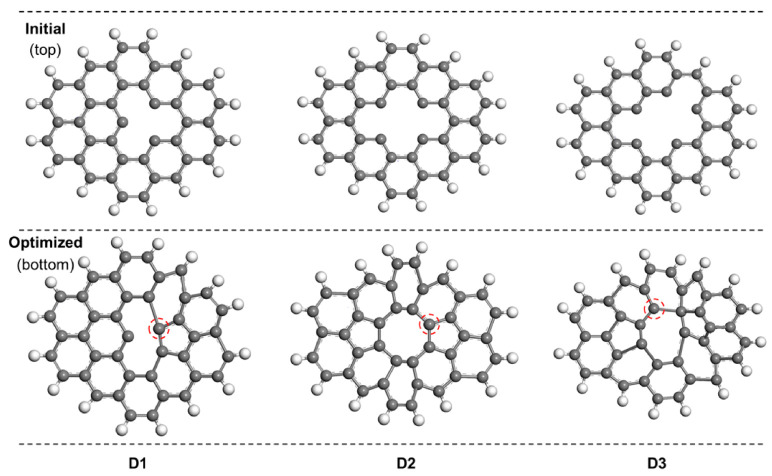
The structures for three typical defects (single defect (**D1**), double defects (**D2**) and triple defects (**D3**)) on graphene: The initial structures (**top**) and the optimized structures (**bottom**).

The aforementioned perfect-edged and perfect-central structures are constructed based on perfect graphene structures. However, due to the strong oxidation of the concentrated HNO_3_, the structures of HNO_3_-treated GO (GO-100, GO-110 and GO-120) are more or less damaged. Therefore, other models with different degrees of defects on graphene were considered as well (Figure S2). Here, three typical types (single defect (D1), double defects (D2) and triple defects (D3) were constructed in their initial structures) in Figure 8. The initial structures for the above three typical defects were optimized with B3LYP functional. On the edge of central defect over defected graphene surface, edged -COOH (defected-edged-COOH), -OH (defected-edged-OH), -C=O (defected-edged-C=O) group and -COC (defected-central-COC) group near the edge of the defect were constructed. However, it was found that the defected-edged-C=O group tends to transfer into -COC group automatically, thereby the iodine interaction on this group was not considered [29,35].

**Figure 9:**
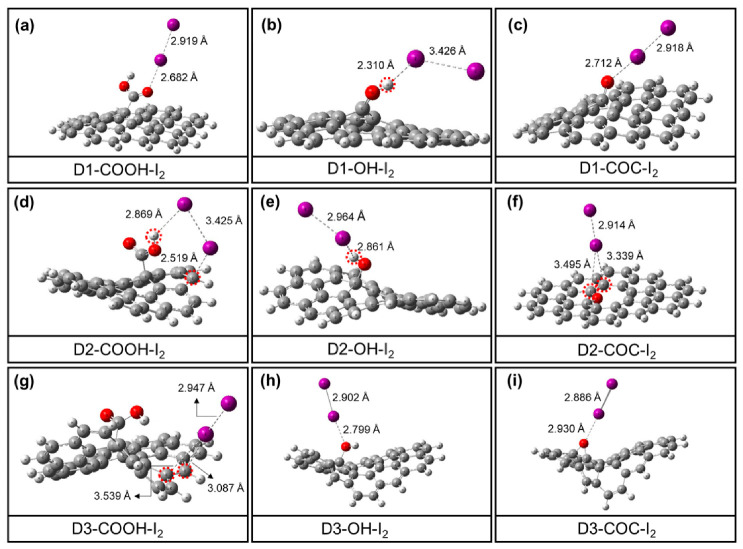
Optimized structures for single-defect structure (**a**–**c**), double-defects structure (**d**–**f**) and triple-defects structure (**g**–**i**) with B3LYP functional.

**Figure 10:**
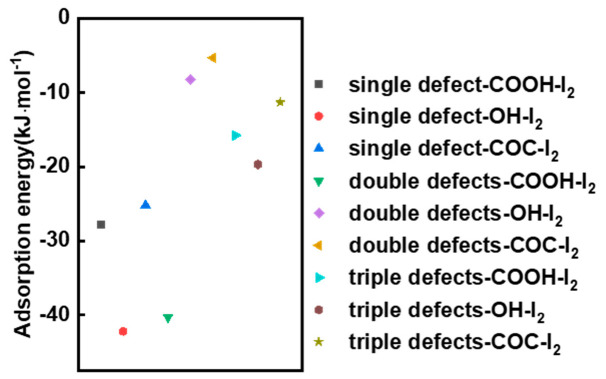
Adsorption energy of the iodine molecules and the oxygen functional groups in single-defect structure (D1), double-defects structure (D2) and triple-defects structure (D3).

**Figure 11:**
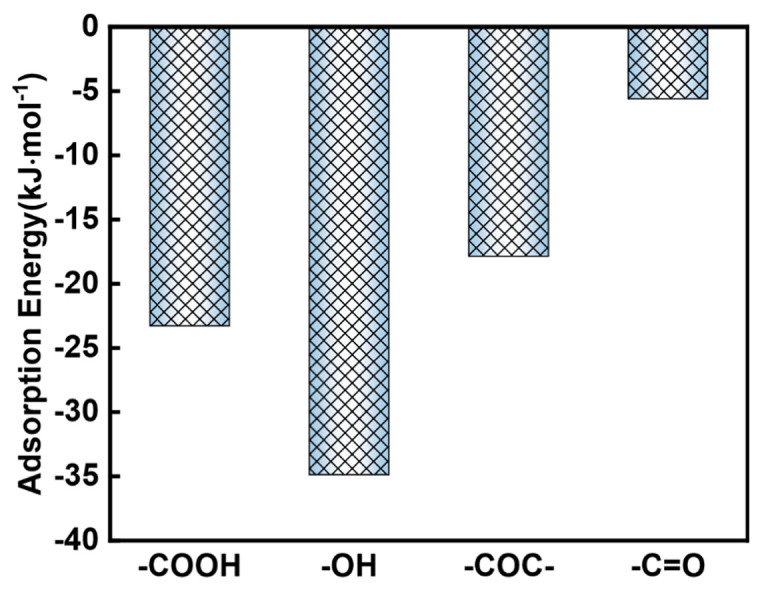
Average adsorption energy of different oxygen functional groups.

The adsorption behavior of iodine by oxygen-containing groups on the graphene surface under different types of defect conditions was investigated next (Figure 9). Compared to the I-I bond lengths in iodine molecules alone (Figure 6a), it is evident that when iodine molecules interact with oxygen-containing groups on the surface of graphene of different defect types, the I-I bond lengths in iodine molecules are also increased to some extent, which is similar to what happens in the perfect model. This also indicates that the presence of oxygen-containing groups has a slight pull on the iodine molecule. Similarly, in the defected models, the oxygen functional groups on the graphene surface, such as the carboxyl groups in double defects and triple defects structures, can activate the nearby carbon and hydrogen atoms (in red circles) to form adsorption active sites. In addition to this, the average bond length between -OH and iodine molecules was found to be the shortest regardless of the defected structure. This is also in agreement with the experimental results in GO-120 sample. The adsorption energies of different oxygen-containing functional groups on the surface of different defected graphene for iodine molecules were further calculated. As shown in Figure 10, the adsorption energies of all the oxygen-containing defected graphene for iodine molecules are located in the range of −5.3 kJ·mol^−1^ (double defects-COC) to −42.2 kJ·mol^−1^ (single defect-OH). In general, the -OH group tend to have a larger adsorption energy averagely (Figure 11). This explains why GO-120 displays the highest iodine uptake capacity. Such results further indicate that the introduction of oxygen-containing functional groups on the surface of both the perfect and defective models allows the oxygen-containing functional groups to interact directly with the iodine molecules, as well as to activate the surrounding carbon atoms. Due to the synergistic promotion of these two interactions, the adsorption capacity of graphene to iodine molecules is increased.

## 4. Conclusions

In conclusion, a series of graphene oxide nanosheets with rich surface oxygen functional groups were prepared as solid adsorbents for radioactive iodine adsorption after a simple nitric acid treatment. A positive correlation was observed between the iodine uptake capacity and the oxygen content and the diversities of oxygen functional groups according to the experimental results. The sample GO-120, which possesses an ultrahigh oxygen content of up to 52.04 wt.% and most abundant oxygen functional groups, displays the highest iodine uptake capacity. To further prove the effects of oxygen on iodine adsorption, the density functional theory calculation was also carried out. Both perfect and defected surfaces were considered on the surface of graphene to match the real experimental material. The DFT calculation results show that the presence of oxygen functional groups enhances the interaction between the iodine molecules and adsorbents. There is a covalent bond forming during the interaction between iodine molecule and O atoms or C/H atoms activated by nearby oxygen functional groups. Among the four kinds of oxygen functional groups, -OH-I_2_ system displays the highest adsorption energy averagely (−34.88 kJ·mol^−1^) while the adsorption energy of -COOH and -COC- groups is relatively lower (−23.28 kJ·mol^−1^ and −17.87 kJ·mol^−1^, respectively). The -C=O group has the least obvious influence for the average adsorption energy is only −5.6 kJ·mol^−1^. The effects of different oxygen functional groups are systematically investigated in this work. which opens up a new research direction for the design of carbon materials used as iodine adsorbents in the future.

## Figures and Tables

**Table 1:** Contents of C and O of different samples.

Sample	C (wt.%)	O (wt.%)	C/O ^1^
GO	53.14	46.86	1.51
GO-100	49.40	50.60	1.30
GO-110	48.82	51.18	1.27
GO-120	47.97	52.04	1.23

^1^ Atomic ratio.

## Supplementary Materials

The following are available online at https://www.mdpi.com/1996-1944/13/24/5770/s1, Table S1: Adsorption energy values of different oxygen-containing group, Table S2: Bond distances of different oxygen functional groups, Figure S1: Distinct structures of oxygen functional groups on perfect surface of graphene. The carbon atoms activated by neighboring oxygen functional groups are within red circles, Figure S2: Distinct structures of oxygen functional groups on three kinds of defected surfaces of graphene. The carbon atoms activated by neighboring oxygen functional groups are within red circles.
