# Supplementary material for: The Effects of Oxygen Functional Groups on Graphene Oxide on the Efficient Adsorption of Radioactive Iodine
Source: Materials (Basel). 2020 Dec 17;13(24):5770. doi: 10.3390/ma13245770 (PMC7766158; doi:10.3390/ma13245770)
Supplement: Supplementary file 1 [file materials-13-05770-s001.pdf]

materials

Supporting Information

The Effects of Oxygen Functional Groups on Graphene Oxide on The Efficient Adsorption of Radioactive Iodine

Qian Zhang [1], Yangyang Gao [1], Zhanglian Xu [1,*], Sheng Wang [1,*], Hisayoshi Kobayashi [2] and Jie Wang [1,*]

[1] Shaanxi Key Laboratory of Advanced Nuclear Energy and Technology and Shaanxi Engineering Research Center of Advanced Nuclear Energy, School of Nuclear Science and Technology, Xi'an Jiaotong University, Xi'an, Shaanxi, 710049, P. R. China; imzhangqian@stu.xjtu.edu.cn (Q.Z.); gyy2019@stu.xjtu.edu.cn (Y.G.)

[2] Department of Chemistry and Materials Technology, Kyoto Institute of Technology, Matsugasaju, Sakyo-ku, Kyoto, 606-8585, Japan; hisabbit@yahoo.co.jp

* Correspondence: xuzhanglian@xjtu.edu.cn (Z.X.); shengwang@xjtu.edu.cn (S.W.); wangjie1@xjtu.edu.cn (J.W.) Tel.: +86-177-7897-3029 (Z.X.); +86-137-5707-3588 (S.W.); +86-189-9161-2872 (J.W.)

Table S1. Adsorption energy values of different oxygen-containing groups.

System with B3LYP		ΔE(kJ/mol)
graphene	$C_{24}H_{12}$-I_2	0.3
perfect-edged-COOH-I_2	$C_{42}H_{15}$-COOH-I_2	−9.2
perfect-edged-OH-I_2	$C_{42}H_{15}$-OH-I_2	−22.9
perfect-edged-C=O-I_2	$C_{42}H_{15}$-O-I_2	−5.6
perfect-central-OH-I_2	$C_{42}H_{16}$-OH-I_2	−81.4
perfect-central-COC-I_2	$C_{42}H_{16}$-O-I_2	−21.8
single defect-COOH-I_2	$C_{41}H_{16}$COOH-I_2	−27.8
single defect-OH-I_2	$C_{41}H_{16}$OH-I_2	−42.2
single defect-COC-I_2	$C_{41}H_{16}$O-I_2	−25.2
double defects-COOH-I_2	$C_{40}H_{16}$COOH-I_2	−40.3
double defects-OH-I_2	$C_{40}H_{16}$OH-I_2	−8.2
double defects-COC-I_2	$C_{40}H_{16}$O-I_2	−5.3
triple defects-COOH-I_2	$C_{39}H_{16}$COOH-I_2	−15.8
triple defects-OH-I_2	$C_{39}H_{16}$OH-I_2	−19.7
triple defects-COC-I_2	$C_{39}H_{16}$O-I_2	−11.3

Table S2. Bond distances of different oxygen-containing groups.

System with B3LYP	Bond Distance[Å]		
	I-C	I-O	I-H
graphene-I_2	8.431	—	—
perfect-edged-COOH-I_2	3.202	—	—
perfect-edged-OH-I_2	2.970	—	—
perfect-edged-C=O-I_2	3.297	—	—
perfect-central-OH-I_2	—	2.107	—
perfect-central-COC-I_2	—	2.720	—
single defect-COOH-I_2	—	2.682	—
single defect-OH-I_2	—	—	2.310
single defect-COC-I_2	—	2.712	—
double defects -COOH-I_2	2.519		2.869
double defects-OH-I_2	—	—	2.861
double defects-COC-I_2	3.339/3.495	—	—
triple defects-COOH-I_2	3.087/3.539	—	—
triple defects-OH-I_2	—	2.799	—
triple defects-COC-I_2	—	2.930	—

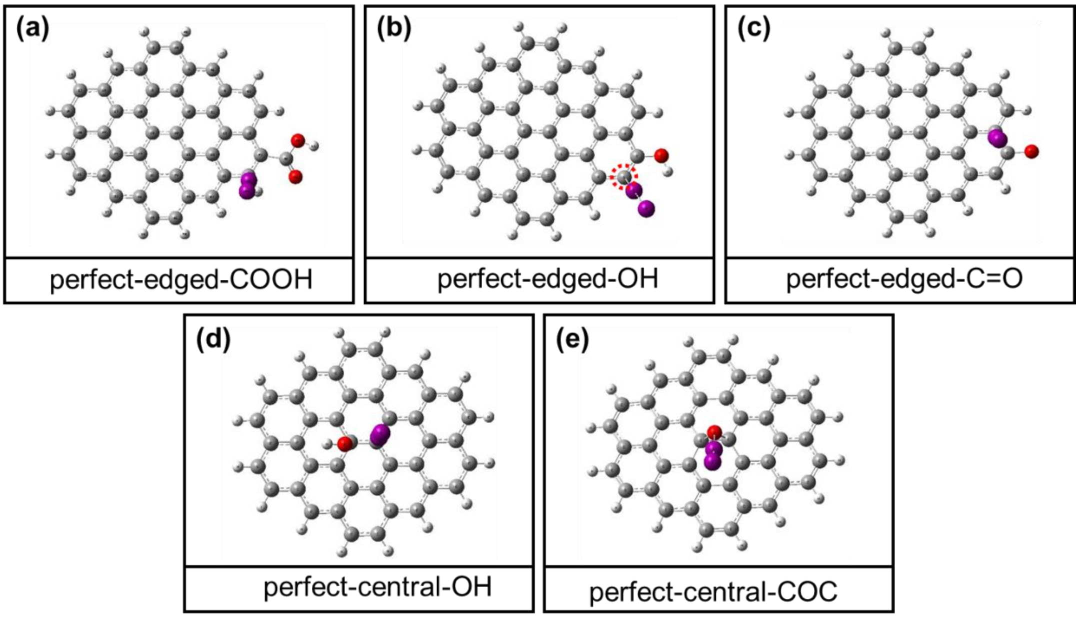

Figure S1. Distinct structures of oxygen functional groups on perfect surface of graphene. The carbon atoms activated by neighboring oxygen functional groups are within red circles.

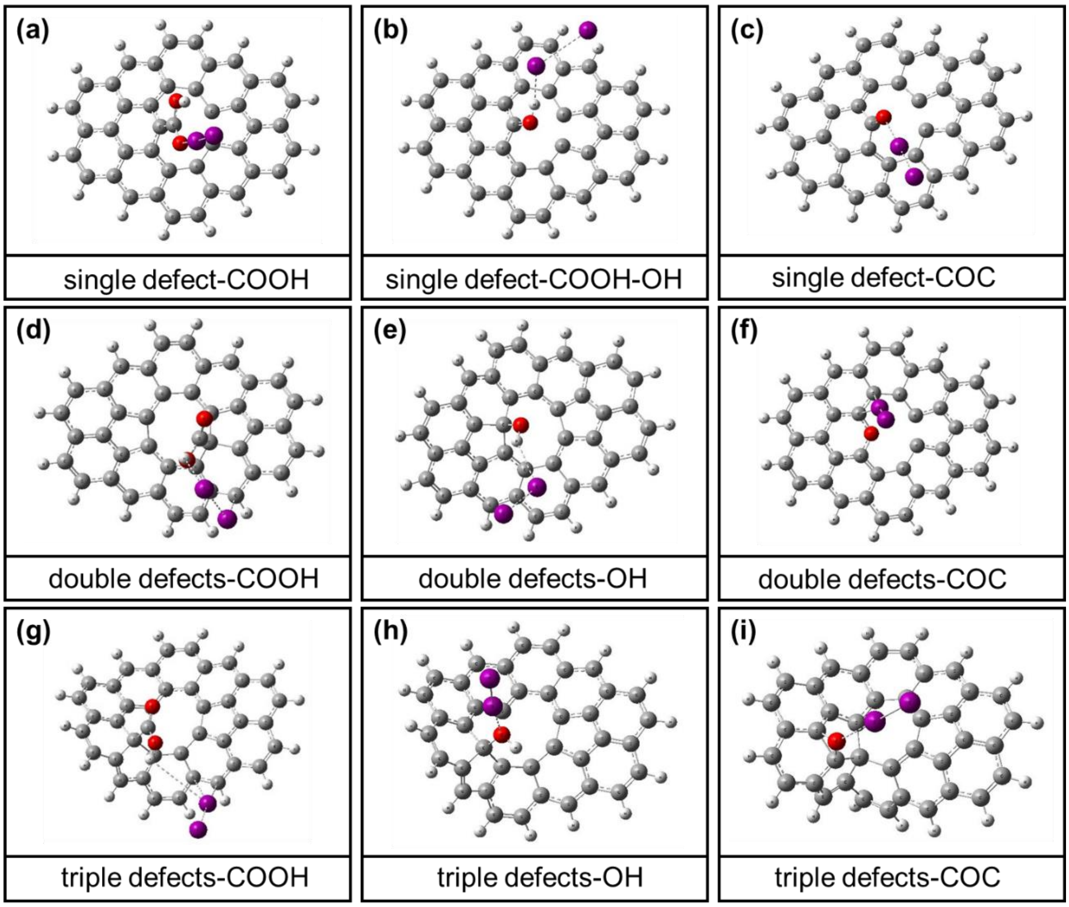

Figure S2. Distinct structures of oxygen functional groups on three kinds of defected surfaces of graphene. The carbon atoms activated by neighboring oxygen functional groups are within red circles.
